# Comparison of microbial taxonomic and functional shift pattern along contamination gradient

**DOI:** 10.1186/s12866-016-0731-6

**Published:** 2016-06-14

**Authors:** Youhua Ren, Jiaojiao Niu, Wenkun Huang, Deliang Peng, Yunhua Xiao, Xian Zhang, Yili Liang, Xueduan Liu, Huaqun Yin

**Affiliations:** School of Minerals Processing and Bioengineering, Central South University, Changsha, 410083 China; Key laboratory of Biometallurgy, Ministry of Education, Changsha, 410083 China; College of Food Science and Technology, Hunan Agricultural University, Changsha, 410083 China; State Key Laboratory for Biology of Plant Diseases and Insect Pests, Institute of Plant Protection, Chinese Academy of Agricultural Sciences, Beijing, 100193 China

**Keywords:** Heavy metal contamination, Microbial remediation, Taxonomic and functional composition, Null model test

## Abstract

**Background:**

The interaction mechanism between microbial communities and environment is a key issue in microbial ecology. Microbial communities usually change significantly under environmental stress, which has been studied both phylogenetically and functionally, however which method is more effective in assessing the relationship between microbial communities shift and environmental changes still remains controversial.

**Results:**

By comparing the microbial taxonomic and functional shift pattern along heavy metal contamination gradient, we found that both sedimentary composition and function shifted significantly along contamination gradient. For example, the relative abundance of *Geobacter* and *Fusibacter* decreased along contamination gradient (from high to low), while *Janthinobacterium* and *Arthrobacter* increased their abundances. Most genes involved in heavy metal resistance (e.g., *metc*, *aoxb* and *mer*) showed higher intensity in sites with higher concentration of heavy metals. Comparing the two shift patterns, there were correlations between them, because functional and phylogenetic β-diversities were significantly correlated, and many heavy metal resistance genes were derived from *Geobacter*, explaining their high abundance in heavily contaminated sites. However, there was a stronger link between functional composition and environmental drivers, while stochasticity played an important role in formation and succession of phylogenetic composition demonstrated by null model test.

**Conclusions:**

Overall our research suggested that the responses of functional traits depended more on environmental changes, while stochasticity played an important role in formation and succession of phylogenetic composition for microbial communities. So profiling microbial functional composition seems more appropriate to study the relationship between microbial communities and environment, as well as explore the adaptation and remediation mechanism of microbial communities to heavy metal contamination.

**Electronic supplementary material:**

The online version of this article (doi:10.1186/s12866-016-0731-6) contains supplementary material, which is available to authorized users.

## Background

Investigating microbial community composition, structure and function, as well as their response to environmental changes are key issues in microbial ecology. Nowadays, microbial communities have been studied both phylogenetically and functionally. Of them, 16S rRNA gene amplicons sequencing is the most effective and popular method to profile microbial community composition, and many uncultured species of nature environments have been identified [1, 2], but it provides little information about the metabolic potential of these species. GeoChip is a comprehensive microarray for investigating microbial community function, also provides direct linkages of microbial genes to ecosystem processes and functions [3]. However, the relationship between community composition and function still remains controversial, which also makes it difficult to evaluate the interactions between microorganisms and environment [4, 5]. For example, a study of soil microbial communities in forest showed that microbial community structure and functional gene diversity were significantly linked to soil and plant factors, but some environmental factors correlated to community structure were not related to its function, such as temperature and tree diameter [6]. Exploring the relationship between microbial community structure and function would help explaining such inconsistency.

Basically, microbial phylogeny and function are strongly correlated, and phylogenetic trees based on 16S usually closely resemble clusters obtained on the basis of shared gene content. Therefore, researchers often infer properties of uncultured organisms from cultured relatives [7]. For example, using 16S information, PICRUSt recaptures key findings from the Human Microbiome Project and accurately predicts the abundance of gene families in host-associated and environmental communities [8]. However, widespread cases of horizontal gene transfer impair the consistency between taxonomic and functional composition. And it was recently suggested that functional traits are more valuable to understand bacterial community assembly and explain shifts in microbial community composition across environmental gradients [9, 10], probably because the adaptation and ecological niche of microbial population was more directly determined by their functional composition.

Microbial communities usually change extensively under environmental stress, which provides us an opportunity to explore the correlation and difference between microbial phylogenetic and functional shift pattern. Recently, heavy metals such as mercury (Hg), chromium (Cr), lead (Pb), manganese (Mn), and arsenic (As) have induced serious diseases or even death of organisms through contaminated waters or soils, although heavy metals in trace amount are beneficial even significant to organisms [11–13]. The investigation of microbial populations distribution along heavy metal contamination gradients across spatial scales will help elucidate how natural communities respond to environmental changes [14]. Many studies on the issue have been focused on functional and phylogenetical analyses. For example, in highly heavy metal contaminated sites, an overall lower gene diversity but higher abundance for specific functional genes, such as heavy metal homeostasis genes and sulfate-reducing genes were observed [11, 15, 16], and the dominant microbial groups included *α-Proteobacteria*, *β-Proteobacteria* and *Firmicutes* [17, 18].

In this study, we attempted to address two core issues (i) the taxonomic and functional shift pattern of sedimentary microbial communities to heavy metal contamination; and (ii) correlation and difference among the two shift patterns. To explore the taxonomic and functional response of microbial communities to heavy metal contamination, 12 sedimentary samples were taken from three sites in the Xiangjiang River with a gradient of contaminant levels (described before [19]), and analyzed by GeoChip 5.0 and 16S rRNA gene amplicons sequencing. The study provides us an insight into the shift pattern of microbial communities to heavy metal contamination, and demonstrates that functional profiling microbial communities is more effective in examining the interaction between microorganisms and environments.

## Methods

### Sample description

Samples were collected from sediment of Xiangjiang River (Hunan, China), as previous described [19]. In this study, we choose three groups of samples with different distance from drain outlet, 500 m, 1000 m, and 1500 m, separately. Geochemical properties of each sample were measured. The composition of heavy metals including Hg, As, Cr, Pb, Mn, cobalt (Co), cadmium (Cd), nickel (Ni), copper (Cu) and zinc (Zn) in the sediments was analyzed by ICP-AES [20]. Total sedimentary organic nitrogen (N) was quantified by Kjeldahl distillation [21]. The amount of total sedimentary organic carbon (C) was analyzed by potassium dichromate oxidation-ferrous sulphate titrimetry [22].

### Illumina sequencing, GeoChip analysis and data processing

DNA was extracted using a TIANamp Bacterial DNA Kit (MO BIO Laboratories, Inc., Carlsbad, CA). The V4 region of the 16S rRNA genes was amplified with the primer pair 515 F (5’-GTGCCAGCMGCCGCGGTAA-3’) and 806R (5’- GGACTACHVGGGTWTCTAAT-3’). Sample libraries were generated from purified PCR products. The MiSeq 500 cycles kit was used for 2x250 bp paired-ends sequencing on MiSeq machine (Illumina, San Diego, CA). Sequences with perfect matches to barcodes were split to sample libraries, and trimmed. OTU clustering was performed through UCLUST at 97 % similarity level [23], and taxonomic assignment was through the RDP classifier [24] with a minimal 50 % confidence estimate. The above steps were conducted through the Galaxy pipeline (http://zhoulab5.rccc.ou.edu/) developed by Qin el al. Subsequent analyses were performed in R [25]. Finally, samples were rarefied at 13,000 sequences per sample. All the 16S rRNA sequences were deposited in GenBank database and the accession number were KP784842 - KP788032.

For each sample, microbial community DNA was extracted and purified as described previously [15, 26]. Amplified DNA was labeled and hybridized with GeoChip 5.0, which is a powerful tool to study the functional diversity, composition, structure and metabolic potential of microbial communities [6]. All GeoChip 5.0 hybridization data are available at the Institute for Environmental Genomics, University of Oklahoma (http://ieg.ou.edu/). The hybridized GeoChip 5.0 was analyzed as previously described [27]. Software TMEV was used for hierarchical cluster analysis of sequencing and GeoChip data. Statistical differences between the functional microbial communities from the different sites were analyzed by analysis of variance (ANOVA).

### Statistical analyses

Partial least squares path modeling (PLSPM) is a powerful structural equation modeling technique, which is used to elucidate the complex relationship among microbial community composition, structure and function of three groups of samples. Before performed in R v. 2.6.1 with the package *plspm* [25], principal component analysis (PCA) was conducted for 16S rRNA gene sequencing data, GeoChip hybridization data and environmental data respectively. Then PC1 and PC2 values were used for PLSPM. And α-diversity value could be used for PLSPM directly. Taxonomic composition and functional gene diversity was calculated using Shannon-Weiner’s H′ and evenness. Difference among three groups of microbial communities in composition and function was evaluated using dissimilarity test respectively. Mantel test was used to calculate correlations between microbial community diversity and environmental attributes [28]. Null model analysis which assumes that a community is not structured by species interactions, was performed according to the method described by Zhou et al [29]. In order to determine whether species/gene compositional differences among sites were caused by the forces causing communities to be different from the expectations by random chance or not, the permutational analysis of multivariate dispersions (PERMDISP) was used to test the significance of the differences of the sedimentary microbial communities of each group from null model expectations [30]. All the analyses were performed online (http://ieg.ou.edu/).

## Results

### Geochemical parameters

Geochemical properties of samples were significantly (*p* < 0.05) different among three groups (Additional file 1: Table S1). The sample group nearest (500 m) to drain outlet had the highest concentration of heavy metals and organic C (1.51 ppm) and N (16.68 ppm), so we defined the group as Group H. Almost all the heavy metals detected in this study were most abundant in Group H, except for Pb and Zn. On the contrary, the sampling site farthest (1500 m) from drain outlet had the lowest concentration of heavy metals, thus was defined as Group L. The concentrations of Hg (0.18 ppm), Cd (3.0 ppm), Cu (34 ppm) and Zn (158 ppm) were the lowest in Group L. And the sample group with a sampling site between Group H and Group L was defined as Group M, which was about 1000 m away from the drain outlet. Group M had a moderate content of heavy metals, with similar Pb (103 and 124 ppm) and Zn (346 and 496 ppm) content to Group H, but similar As (70 and 73 ppm), Co (12.1 and 12.8 ppm), Cr (57 and 62 ppm), Ni (30.2 and 32.7 ppm) and Mn (788 and 1476 ppm) content to Group L.

### Shift patterns of taxonomic and functional community composition

**Fig. 1:**
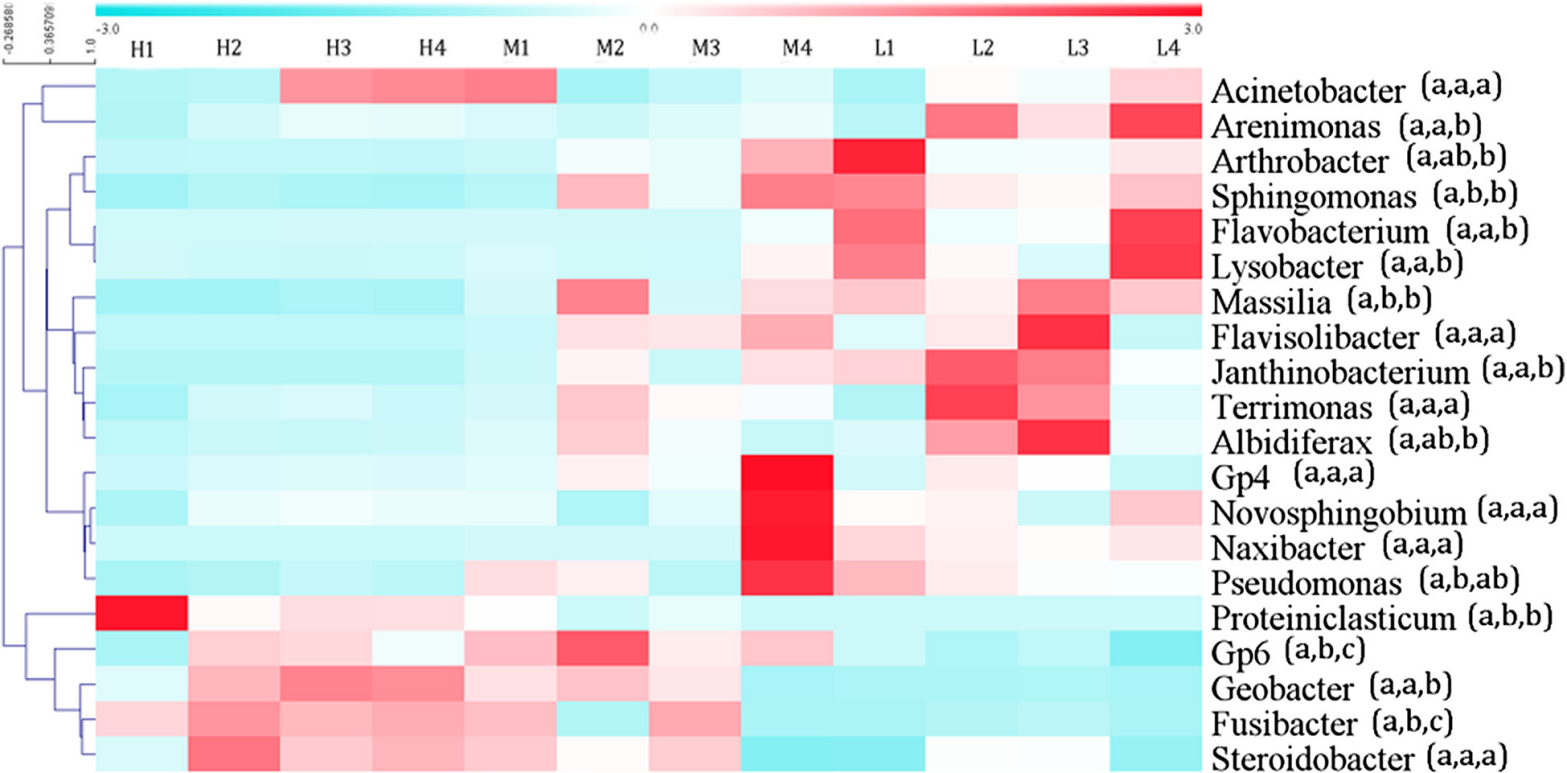
Hierarchical cluster analysis of sequencing data of 12 samples at the genus level. Relative abundances of microbial genera were standardized before hierarchical clustering. Significant differences (*P* < 0.05) among three groups are indicated by alphabetic letters

All three groups of communities were mainly composed of *Fusibacter* (1.42 to 35.21 %) and *Janthinobacterium* (0.07 to 18.35 %), followed by *Proteiniclasticum* (0.02 to 12.96 %), *Acinetobacter* (3.15 to 4.44 %) and *Massilia* (0.14 to 4.93 %). In addition, 14.62–17.81 % OTUs could not be classified into any known genus. Both Shannon diversity and Pielou evenness indices (3.80 and 0.56 respectively) were significantly (*p* < 0.05) lower in Group H than other two groups (Additional file 1: Table S2). Dissimilarity test showed that three groups of microbial communities were significantly (*p* < 0.1) different from each other in composition and structure (Table 1). Hierarchical cluster analysis of sequencing data at the genus level (relative abundance > 1 %) showed that sedimental microbial community composition and structure shifted substantially along river (Fig. 1). For example, *Fusibacter*, *Geobacter*, *Gp6* and *Proteiniclasticum* were significantly (*p* < 0.05) more abundant in heavily contaminated samples, while Group L had more *Janthinobacterium*, *Arthrobacter*, *Sphingomonas* and *Flavobacterium*. Especially, the relative abundance of *Fusibacter* decreased along the heavy metal contamination gradient (from high to low), while *Janthinobacterium* increased its relative abundance along the gradient.

**Table 1:** Dissimilarity test of three groups of microbial communities in phylogenetic composition

		Group H	Group M
Group M	Distance	0.459	
	Significance	0.028	
Group L	Distance	0.41	0.56
	Significance	0.039	0.0919

**Fig. 2:**
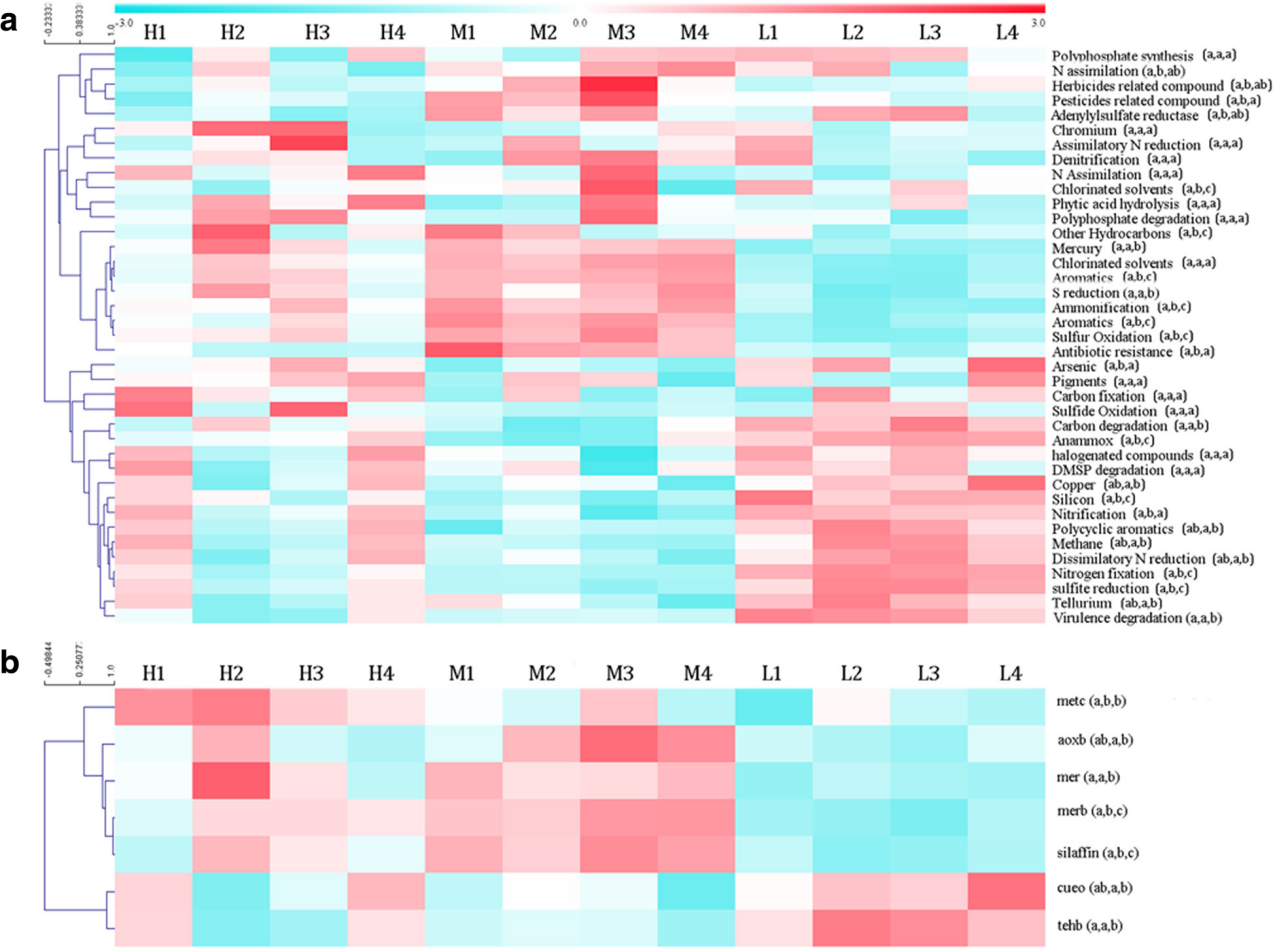
Hierarchical cluster analysis of GeoChip hybridization data of 12 samples (**a**) and hierarchical cluster analysis of seven genes involved in heavy metal resistance (**b**). Relative intensities of genes were standardized before hierarchical clustering. Significant differences (*P* < 0.05) among three groups are indicated by alphabetic letters

A total of 29,439 gene variants were detected in GeoChip. They included gene groups of C, N, phosphorus (P), and sulfur (S) cycling, metal homeostasis, organic remediation and secondary metabolism. Shannon diversity was the highest in Group L (10.20) and lowest in Group M (9.93), and Pielou evenness was lower in Group L than the other two groups (Additional file 1: Table S2). More specifically, 355 genes could be assigned into 39 subcategories. Dissimilarity test showed that three groups of microbial communities were significantly (*p* < 0.05) different from each other in function (Table 2). Hierarchical cluster analysis at the subcategory level showed that 14 gene subcategories showed significantly (*p* < 0.05) stronger intensity in Group H and Group M, such as gene subcategories related to herbicides related compound, pesticides related compound and chlorinated solvents. And 13 gene subcategories had higher abundance in Group L (e.g., nitrogen fixation, carbon fixation and degradation) (Fig. 2). Most genes involved in heavy metal resistance showed higher abundance in Group H and Group M, including *aoxb*, *metc*, *mer* and *merb*. Of them, *aoxb* is related to As resistance, and *metc*, *mer* and *merb* are related to Hg resistance. Besides, gene *cueo* (Cu resistance) and *tehb* (Tellurium resistance) showed no significant difference between Group H and Group M in abundance, but higher relative abundance in Group L than Group M.

**Table 2:** Dissimilarity test of three groups of microbial communities in functional composition

		Group H	Group M
Group M	Distance	0.093	
	Significance	0.028	
Group L	Distance	0.086	0.069
	Significance	0.026	0.034

### Comparison of taxonomic and functional shift pattern

**Fig. 3:**
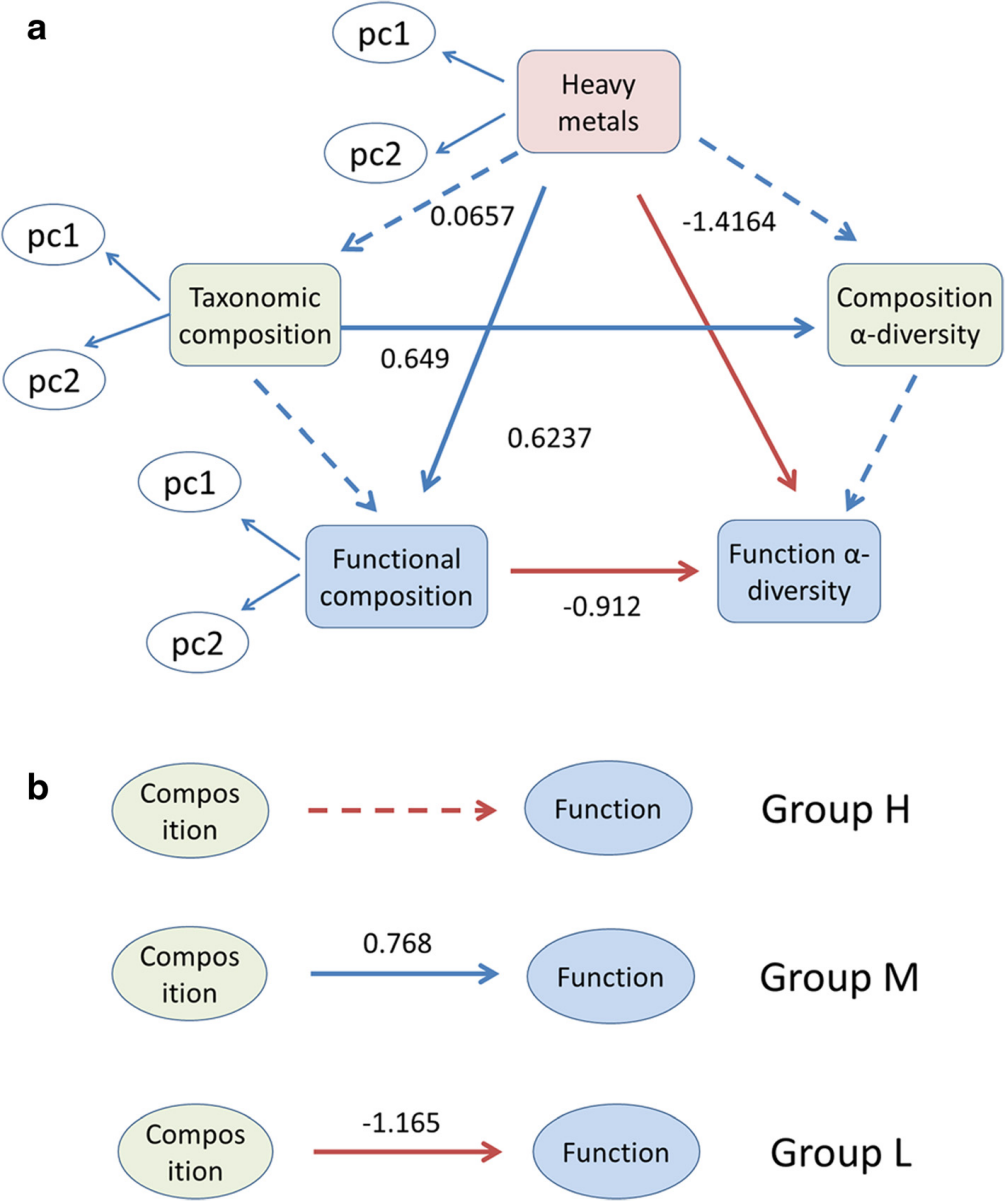
Partial least squares path modeling (PLSPM) about correlations among sediment properties (C&N and heavy metals), microbial community composition (abundance and diversity) and function (intensity and diversity) (**a**), as well as between microbial community composition and function of Group H, Group M and Group L respectively (**b**). Only significant (*p* < 0.01) correlations are indicated with solid line

Two PLSPMs were performed to profile the relationship among sediment properties, microbial community taxonomic and functional composition. Goodness of fit (Gof) value was 0.5885 and 0.6231 for each model, bigger than 0.35, indicating that the two PLSPMs were reliable. Results showed that environmental factors were significantly correlated to microbial function, but not community composition (Fig. 3a). PLSPM also indicated the correlation between community composition and function, especially in communities of Group M and Group L (Fig. 3b).

Generally, a significant correlation (*r* = 0.711, *p* = 0.001) between phylogenetic β-diversity and functional β-diversity was observed. Although not all the microbial populations detected by 16S rRNA gene sequencing had their genes detected in GeoChip, there were still 10 microbial genera (relative abundance > 1 %) whose genes were detected by GeoChip. For example, 71 gene variants of *Geobacter*, 24 gene variants of *Janthinobacterium* and 211 gene variants of *Arthrobacter* were detected. More importantly, of all gene variants belonging to *Geobacter* 36.62 % of them were involved in heavy metal resistance, but only 4.17 % of *Janthinobacterium* genes were related to heavy metal resistance, indicating their different potential in adapting heavy metal contamination (Additional file 1: Table S3).

The two shift patterns showed difference in their relationship with environment. Mantel test revealed that only a small proportion (17.50 %) of microbial genera were significantly (*p* < 0.05) correlated with environmental factors, while most of (66.04 %) the functional gene groups were correlated to sedimentary properties (Additional file 1: Table S4). For example, Hg had impact on eight microbial genera (e.g., *Fusibacter*, *Geobacter*, *Gp6* and *Janthinobacterium*) but up to 30 functional gene groups, such as nitrification, nitrogen fixation, carbon fixation and carbon degradation. Furthermore, null model analysis was performed to investigate the effect of stochastic process on assembly and succession of microbial communities, based on sequencing data and GeoChip hybridization data respectively. PERMDISP test revealed that the observed β-diversity was indistinguishable from the null expectation for all three groups of communities at phylogenetic level, whereas the observed β-diversity was significantly (*p* < 0.05) different from the null random expectations for the sedimentary communities in Group H and Group M at functional level (Table 3). It indicated that stochasticity played an important role in assembly and succession of phylogenetic composition while functional composition was controlled more by deterministic process (e.g., environmental stress).

**Table 3:** Significance test of the differences of centroids between the sedimentary microbial communities and null model simulations across different contamination gradient

	Group	Centroid of actual communities	Centroid of the null model	*F*	*P*
Taxonomic composition	H	0.2509904	0.3284943	3.359766	0.116508
	M	0.3659146	0.2926673	2.052963	0.201888
	L	0.3185871	0.3498911	0.61719	0.46198
Functional composition	H	0.08969783	0.17308444	6.669926	**0.041623**
	M	0.06876982	0.23208962	106.9815	**4.78E–05**
	L	0.05090288	0.06295226	0.303056	0.601851

Permutational analysis of multivariate dispersions (PERMDISP) was used. P values < 0.05 in bold

## Discussion

The interaction mechanism between microbial communities and environment is a key issue in microbial ecology. Microbial communities usually change significantly under environmental stress, which provides us an opportunity to study the microorganism-environment interaction mechanism. Here, by investigating how sedimentary microbial communities shifted along heavy metal contamination gradient both phylogenetically and functionally, we aimed to identify an effective method to profile the relationship between microbial communities and environment, and reveal the adaptation mechanism of sedimentary microbial communities to heavy metal contamination.

### Taxonomic and functional shift pattern along contamination gradient

Generally, both phylogenetic and functional composition of microbial communities shifted significantly along contamination gradient. Previous studies showed that heavy metals would decrease the diversity of microbial community [15, 31], and we observed the same tendency as microbial communities had the lowest Shannon diversity index in H Group. All kinds of microbial populations were clustered into three major categories, according to their abundance pattern in three sites. They included microbial genera positively related to heavy metal contamination, negatively related to heavy metal contamination and showed no obvious change along contamination gradient. For example, *Fusibacter* and *Geobacter* were positively correlated to contamination, while *Janthinobacterium* showed a negative correlation. A larger percent of *Fusibacter* (35.21 %) were detected in Group H, probably because many *Fusibacter* species could reduce sulfur or thiosulfate [32, 33], which usually are of high metal resistance and play important roles in heavy metal bioremediation. *Geobacter* were more abundant in Group H and Group L, mainly resulting from their metal ion reduction ability and potential for use in bioremediation of radioactive metals [34, 35]. On the contrary, *Janthinobacterium* is an important genus of *Betaproteobacteria* [36], and it is found to be susceptible to heavy metals, such as Ag, Cu, Hg, Pb and Ni [37]. In this study, the relative abundance of *Janthinobacterium* decreased significantly in highly contaminated samples, consistent with previous studies.

Meanwhile, functional composition of microbial communities also shifted along contamination gradient. It has been reported that prolonged exposure to high concentrations of heavy metals resulted in a significant loss of metabolic diversity [38]. Although a decrease in metabolic diversity hasn’t been detected in heavily contaminated sites in this study, which might resulted from their long-term adaptation to contaminated environment [39], lots of genes involved in carbon and nitrogen metabolism showed a decrease in intensity, such as *nifh* and *amyA*, involved in nitrogen fixation and carbon degradation respectively. The results were supported by previous studies in which heavy metal pollution produced a dramatic reduce in nutrient metabolism [40, 41]. However, microorganisms have several mechanisms to survive heavy metal contamination, such as discharging toxic metals and enzymatic conversion [42, 43]. In this way, high intensity of heavy metal resistance genes was usually observed in heavy contaminated sites. Here, we detected higher abundance of heavy metal resistance genes (e.g., *metc*, *aoxb*, *mer*, *merb* and *silaffin*) in Group H while lower abundance in Group L, which was a vital part in the functional shift process of sedimentary microbial communities.

### Taxonomic and functional compositions were correlated

Furthermore, we compared the phylogenetic and functional shift pattern in order to identify an effective method to study the interactions between microbial communities and environments as well as the adaptation mechanism of microorganisms to heavy metal contamination. Comparing the two shift patterns, correlation between them is one important point, because microbial populations respond to environment through their functional genes. For example, most of microorganisms in AMD are capable of oxidizing Fe or S, so we could approximately predict the Fe or S oxidation potential of a community based on 16S rRNA gene sequencing data [44]. Here, genes related to heavy metal resistance were derived mainly from *Actinobacteria*, *Gammaproteobacteria*, *Alphaproteobacteria* and *Betaproteobacteria*, which were also the main classes in sedimentary microbial communities. However, not all the gene variants detected in GeoChip could be assigned to the specific microbial populations identified by sequencing, because probes in microarray are usually designed before knowing the microbial community composition [3]. Nevertheless, the different potential in heavy metal resistance of *Geobacter* and *Janthinobacterium* detected by Geochip, was consistent with their abundance pattern in three contamination sites, demonstrating the correlation between taxonomic and functional shift pattern of microbial populations. Because of the limitation of GeoChip, a metagenomic insight into the microbial communities is needed to profile the relationship between microbial community composition and function in future study. Moreover, we found that functional and phylogenetic β-diversities were significantly (*r* = 0.711, *p* = 0.001) correlated, which was also reported in a previous study [45]. We have acknowledged that microbial species closely related in phylogenetic trees based on 16S usually have similar functional composition, and researchers could almost accurately predict the abundance of gene families in environmental communities based on 16S information [7, 8]. In this way, evidences supported that phylogenetic and functional composition are correlated to a certain extent.

### Functional composition was more environmentally dependent

However, since a broad range of functional variation may occur among closely related organisms, taxonomic distributions are assumed to be ambiguous in assessing the response of microbial communities to environmental changes [46]. And horizontal gene transfer might be the other key factor resulting in the divergence between phylogenetic trees based on 16S information and functional composition [47]. A recent study has documented that specific functions could be widely detected across a variety of taxa or phylogenetic groups [48]. For example, sulphate-reducing bacterial species are widely distributed in various phyla, such as *Deltaproteobacteria*, *Nitrospirae*, *Clostridia* and *Euryarchaeota* [49]. Such differences lead to their different relationship with environment. Researchers reported that functional traits were more reliable in assessing the relationship between microbial communities and ecological processes, as microbial assemblages were better predicted at the functional genes level rather than at taxonomic level [45]. In this study, both mantel test and PLSPM analysis indicated that the responses of functional traits might depend more on environmental changes.

Historically contingent in taxonomic composition indicated that environmental conditions would determine the types of ecological niches available for specific functional groups, while species compositions with similar physiological fitness are stochastically influenced by the history [4]. Particularly, a previous study showed that stochastic processes played important roles in controlling the assembly and succession of the groundwater microbial community [29]. Therefore, we speculated that selection strength, mainly heavy metal contamination stress in this study, shaped and directed the functional shift pattern of sedimentary microbial communities, but their phylogenetic composition had various shift patterns to achieve the same function shift, because similar function genes are widely distributed. For example, heavy metal resistance related genes in this study were derived from 29 microbial phyla, including 57 classes, 107 orders and 502 genera. So each microbial population capable of heavy metal resistance had a chance to become more abundant in heavily contaminated sites in theory. Supporting the hypothesis, we demonstrated that stochasticity played a more significant role in phylogenetic composition than in functional composition of sedimentary microbial communities using null model test.

## Conclusions

Collectively, by comparing the microbial taxonomic and functional shift pattern along heavy metal contamination gradient, the study demonstrated that: (i) the responses of functional traits depended more on environmental changes, and stochasticity played an important role in formation and succession of phylogenetic composition; (ii) taxonomic composition and functional composition were closely correlated, although taxonomically related populations neither sufficiently nor necessarily meant functional similarity. The study is of high significance in future metagenomic research, and also provides us an insight into the adaptation pattern of microbial communities to heavy metal contamination.

## Abbreviations

ANOVA, analysis of variance; As, arsenic; C, carbon; Cd, cadmium; Co, cobalt; Cr, chromium; Cu, copper; Gof, goodness of fit; Hg, mercury; Mn, manganesev; N, nitrogen; Ni, nickel; P, phosphorus; Pb, lead; PCA, principal component analysis; PERMDISP, permutational analysis of multivariate dispersions; PLSPM, partial least squares path modeling; S, sulfur; Zn, zinc

## Additional file

Additional file 1:
**Table S1.** Sediment properties and ANOVA analysis among three groups. **Table S2.** Shannon diversity and Pielou evenness of samples based on sequencing data and Geochip hybridization data respectively. **Table S3.** Correlation between microbial composition and function. **Table S4.** (a) Mantel test of sequencing data with environmental attributes at the genus level; (b) Mantel test of GeoChip hybridization data with environmental attributes. (DOCX 41 kb)
