# Supplementary material for: Comparison of microbial taxonomic and functional shift pattern along contamination gradient
Source: BMC Microbiol. 2016 Jun 14;16:110. doi: 10.1186/s12866-016-0731-6 (PMC4908767; doi:10.1186/s12866-016-0731-6)
Supplement: Additional file 1: — Table S1. Sediment properties and ANOVA analysis among three groups. Table S2. Shannon diversity and Pielou evenness of samples based on sequencing data and Geochip hybridization data respectively. Table S3. Correlation between microbial composition and function. Table S4. (a) Mantel test of sequencing data with environmental attributes at the genus level; (b) Mantel test of GeoChip hybridization data with environmental attributes. (DOCX 41 kb) [file 12866_2016_731_MOESM1_ESM.docx]

**Comparison of** **microbial taxonomic and functional shift pattern along contamination gradient (supporting information)**

Youhua Ren, Jiaojiao Niu, Wenkun Huang, Deliang Peng, Yunhua Xiao, Xian Zhang, Yili Liang, Xueduan Liu, Huaqun Yin

Table S1. Sediment properties and ANOVA analysis among three groups.

|  | Hg | As | Co | Cd | Cr | Ni | Pb | Cu | Mn | Zn | N | C |
| --- | --- | --- | --- | --- | --- | --- | --- | --- | --- | --- | --- | --- |
| Group H | 0.65±0.03 | 248±60 | 23.2±3.7 | 22.1±2.8 | 87±5 | 53.7±5.9 | 124±19 | 69±7 | 2012±399 | 496±55 | 1.51±0.16 | 16.68±1.15 |
| Group M | 0.37±0.12 | 70±15 | 12.8±3.2 | 12.4±6.2 | 62±12 | 32.7±9.4 | 103±12 | 50±12 | 1476±368 | 346±84 | 0.59±0.29 | 8.56±2.88 |
| Group L | 0.18±0.02 | 73±14 | 12.1±0.6 | 3.0±1.4 | 57±0.3 | 30.2±0.8 | 83±23 | 34±2 | 788±136 | 158±22 | 0.82±0.07 | 10.63±1.09 |
| P(ANOVA) | a,b,c | a,b,b | a,b,b | a,b,c | a,b,b | a,b,b | a,a,a | a,b,c | a,b,b | a,a,b | a,b,b | a,b,b |

*All values are in ppm.

*Significant differences (P<0.05) are indicated by alphabetic letters.

Table S2. Shannon diversity and Pielou evenness of samples based on sequencing data and Geochip hybridization data respectively.

| Community | Taxonomic composition | | Functional composition | |
| --- | --- | --- | --- | --- |
| Index | Shannon diversity | Pielou evenness | Shannon diversity | Pielou evenness |
| Group H | 3.80±0.17 | 0.56±0.02 | 10.03±0.04 | 0.99940±0.00001 |
| Group M | 4.74±0.28 | 0.67±0.04 | 9.93±0.02 | 0.99941±0.00001 |
| Group L | 4.44±0.08 | 0.67±0.02 | 10.20±0.02 | 0.99934±0.00003 |
| *P*(ANOVA) | a,b,b | a,b,b | a,b,c | a,a,b |

*Significant differences (P<0.05) among three groups are indicated by alphabetic letters.

Table S3. Correlation between microbial composition and function.

| Genus | Number of genes | Carbon Cycling | Metal Homeostasis | Nitrogen | Organic Remediation | Phosphorus | Sulfur | Virulence |
| --- | --- | --- | --- | --- | --- | --- | --- | --- |
| Geobacter | 71 | 14.08% | 36.62% | 11.27% | 14.08% | 9.86% | 5.63% | 8.45% |
| Janthinobacterium | 24 | 29.17% | 4.17% | 8.33% | 41.67% | 8.33% | 8.33% | 0.00% |
| Arthrobacter | 211 | 48.34% | 11.85% | 5.69% | 30.81% | 3.32% | 0.00% | 0.00% |
| Sphingomonas | 166 | 28.31% | 5.42% | 1.20% | 52.41% | 6.63% | 0.00% | 6.02% |
| Lysobacter | 2 | 50.00% | 0.00% | 0.00% | 50.00% | 0.00% | 0.00% | 0.00% |
| Arthrobacter | 211 | 48.34% | 11.85% | 5.69% | 30.81% | 3.32% | 0.00% | 0.00% |
| Albidiferax | 20 | 42.86% | 14.29% | 4.76% | 19.05% | 4.76% | 4.76% | 4.76% |
| Flavobacterium | 6 | 50.00% | 33.33% | 0.00% | 0.00% | 16.67% | 0.00% | 0.00% |
| Pseudomonas | 831 | 22.14% | 7.10% | 7.22% | 54.51% | 4.69% | 2.65% | 1.68% |
| Acinetobacter | 102 | 28.43% | 11.76% | 4.90% | 43.14% | 6.86% | 0.98% | 3.92% |
| Novosphingobium | 120 | 17.50% | 5.00% | 1.67% | 70.00% | 5.83% | 0.00% | 0.00% |

Table S4(a). Mantel test of sequencing data with environmental attributes at the genus level.

| Genus (>1%) | Hg | As | Co | Cd | Cr | Ni | Pb | Cu | Mn | Zn | N | C |
| --- | --- | --- | --- | --- | --- | --- | --- | --- | --- | --- | --- | --- |
| Acinetobacter | 0.193 | 0.778 | 0.877 | 0.547 | 0.76 | 0.886 | 0.869 | 0.609 | 0.907 | 0.569 | 0.873 | 0.866 |
| Albidiferax | **0.032** | 0.613 | 0.516 | 0.202 | 0.314 | 0.468 | 0.321 | 0.31 | 0.574 | 0.228 | 0.469 | 0.433 |
| Arenimonas | 0.23 | 0.737 | 0.788 | 0.503 | 0.781 | 0.761 | **0.082** | 0.386 | 0.393 | 0.24 | 0.821 | 0.801 |
| Arthrobacter | 0.117 | 0.48 | 0.424 | 0.112 | 0.573 | 0.526 | 0.468 | 0.222 | 0.167 | 0.122 | 0.425 | 0.523 |
| Flavisolibacter | 0.181 | 0.71 | 0.592 | 0.302 | 0.404 | 0.485 | 0.324 | 0.559 | 0.501 | 0.521 | 0.446 | 0.422 |
| Flavobacterium | 0.141 | 0.559 | 0.694 | 0.24 | 0.783 | 0.64 | **0.087** | 0.277 | 0.213 | **0.071** | 0.754 | 0.833 |
| Fusibacter | **0.052** | **0.06** | **0.045** | **0.081** | **0.062** | **0.086** | 0.441 | 0.114 | **0.099** | **0.063** | 0.152 | 0.132 |
| Geobacter | **0.049** | 0.138 | 0.202 | 0.103 | **0.083** | 0.152 | 0.828 | 0.161 | 0.198 | 0.138 | 0.161 | 0.141 |
| Gp4 | 0.303 | 0.933 | 0.477 | **0.089** | 0.386 | 0.398 | 0.504 | **0.06** | 0.139 | 0.22 | 0.591 | 0.688 |
| Gp6 | **0.063** | 0.62 | 0.361 | **0.067** | 0.226 | 0.239 | 0.711 | 0.151 | 0.189 | 0.167 | 0.303 | 0.383 |
| Janthinobacterium | **0.079** | 0.444 | 0.544 | 0.245 | 0.435 | 0.622 | 0.484 | 0.327 | 0.54 | 0.259 | 0.629 | 0.478 |
| Lysobacter | 0.338 | 0.622 | 0.821 | 0.45 | 0.899 | 0.785 | **0.073** | 0.455 | 0.279 | 0.166 | 0.796 | 0.807 |
| Massilia | **0.006** | 0.122 | 0.113 | **0.032** | **0.038** | **0.055** | 0.404 | **0.059** | 0.301 | **0.048** | 0.105 | 0.171 |
| Naxibacter | 0.245 | 0.873 | 0.573 | **0.071** | 0.432 | 0.451 | 0.375 | **0.061** | 0.104 | 0.124 | 0.819 | 0.908 |
| Novosphingobium | 0.47 | 0.651 | 0.568 | 0.122 | 0.51 | 0.48 | 0.275 | 0.105 | 0.128 | 0.184 | 0.684 | 0.82 |
| Proteiniclasticum | **0.007** | **0.093** | **0.086** | 0.188 | 0.167 | 0.145 | 0.317 | **0.024** | 0.518 | **0.094** | **0.041** | 0.112 |
| Pseudomonas | 0.15 | 0.259 | 0.309 | **0.057** | 0.275 | 0.349 | 0.505 | **0.051** | 0.115 | 0.131 | 0.455 | 0.623 |
| Sphingomonas | **0.027** | 0.643 | 0.454 | **0.026** | 0.277 | 0.325 | 0.468 | **0.053** | 0.194 | **0.053** | 0.375 | 0.378 |
| Steroidobacter | 0.172 | 0.162 | 0.19 | 0.117 | 0.299 | 0.249 | 0.24 | 0.197 | **0.012** | **0.041** | 0.632 | 0.732 |
| Terrimonas | 0.162 | 0.817 | 0.636 | 0.486 | 0.493 | 0.608 | 0.474 | 0.512 | 0.668 | 0.435 | 0.557 | 0.387 |

Table S4(b). Mantel test of GeoChip hybridization data with environmental attributes.

|  | Hg | As | Co | Cd | Cr | Ni | Pb | Cu | Mn | Zn | N | C |
| --- | --- | --- | --- | --- | --- | --- | --- | --- | --- | --- | --- | --- |
| Ammonification | **0.081** | 0.201 | **0.047** | **0.055** | **0.009** | **0.01** | 0.482 | **0.043** | **0.012** | **0.031** | **0.048** | **0.092** |
| Anammox | 0.108 | **0.043** | **0.016** | **0.058** | **0.018** | **0.017** | 0.323 | **0.048** | **0.021** | **0.034** | **0.038** | **0.083** |
| Antibiotic resistance | **0.064** | 0.186 | **0.069** | **0.029** | **0.019** | **0.018** | 0.371 | **0.039** | **0.004** | **0.018** | 0.129 | 0.153 |
| Aromatics | **0.075** | 0.211 | **0.056** | **0.041** | **0.015** | **0.009** | 0.416 | **0.043** | **0.01** | **0.039** | **0.06** | **0.089** |
| Arsenic | **0.066** | 0.359 | **0.098** | **0.054** | **0.014** | **0.022** | 0.481 | **0.051** | **0.023** | **0.047** | 0.101 | 0.106 |
| Assimilation | **0.079** | 0.929 | 0.809 | 0.286 | 0.672 | 0.824 | 0.141 | 0.48 | 0.402 | 0.175 | 0.915 | 0.866 |
| Assimilatory N reduction | 0.106 | 0.335 | 0.109 | **0.079** | **0.021** | **0.022** | 0.483 | **0.084** | **0.028** | **0.053** | 0.131 | 0.133 |
| Carbon degradation | **0.074** | 0.292 | **0.075** | **0.049** | **0.016** | **0.016** | 0.363 | **0.055** | **0.009** | **0.04** | **0.089** | **0.093** |
| Carbon fixation | **0.079** | 0.316 | **0.072** | **0.049** | **0.018** | **0.024** | 0.364 | **0.043** | **0.017** | **0.04** | **0.097** | 0.109 |
| Chlorinated solvents | **0.087** | 0.249 | **0.062** | **0.038** | **0.014** | **0.023** | 0.345 | **0.06** | **0.005** | **0.037** | 0.11 | 0.143 |
| Chromium | 0.373 | 0.701 | 0.439 | 0.357 | 0.164 | 0.288 | 0.877 | 0.416 | 0.189 | 0.321 | 0.291 | 0.298 |
| Copper | **0.053** | 0.138 | **0.031** | **0.032** | **0.009** | **0.007** | 0.444 | **0.027** | **0.004** | **0.017** | **0.056** | **0.098** |
| DMSP degradation | 0.173 | 0.488 | 0.215 | **0.096** | **0.054** | **0.091** | 0.451 | 0.104 | **0.039** | **0.075** | 0.226 | 0.266 |
| Denitrification | **0.07** | 0.323 | **0.081** | **0.037** | **0.011** | **0.023** | 0.44 | **0.044** | **0.012** | **0.038** | 0.114 | 0.13 |
| Dissimilatory N reduction | **0.084** | 0.234 | **0.086** | **0.04** | **0.019** | **0.021** | 0.465 | **0.068** | **0.007** | **0.032** | 0.143 | 0.16 |
| Herbicides related compound | **0.075** | 0.267 | **0.078** | **0.041** | **0.027** | **0.02** | 0.462 | **0.049** | **0.009** | **0.044** | **0.083** | 0.119 |
| Mercury | **0.056** | 0.31 | **0.063** | **0.03** | **0.016** | **0.02** | 0.333 | **0.034** | **0.012** | **0.034** | **0.078** | **0.075** |
| Methane | **0.089** | 0.213 | **0.057** | **0.057** | **0.02** | **0.015** | 0.322 | **0.071** | **0.011** | **0.047** | **0.093** | 0.142 |
| N Assimilation | 0.206 | 0.243 | 0.147 | **0.068** | **0.025** | **0.038** | 0.597 | **0.094** | **0.047** | 0.11 | 0.188 | 0.197 |
| Nitrification | **0.037** | 0.279 | **0.062** | **0.024** | **0.007** | **0.007** | 0.485 | **0.033** | **0.03** | **0.017** | **0.036** | **0.038** |
| Nitrogen fixation | **0.071** | 0.241 | **0.053** | **0.036** | **0.023** | **0.026** | 0.308 | **0.052** | **0.01** | **0.032** | **0.063** | **0.086** |
| Other Hydrocarbons | 0.101 | 0.299 | **0.071** | **0.053** | **0.021** | **0.026** | 0.461 | **0.055** | **0.031** | **0.044** | **0.096** | **0.091** |
| Pesticides related compound | **0.097** | 0.415 | 0.122 | **0.052** | **0.025** | **0.033** | 0.38 | **0.095** | **0.006** | **0.03** | 0.171 | 0.14 |
| Phylogenetic | **0.098** | 0.409 | **0.099** | **0.056** | **0.032** | **0.044** | 0.367 | **0.083** | **0.029** | **0.053** | **0.063** | **0.062** |
| Phytic acid hydrolysis | **0.084** | 0.477 | 0.114 | **0.045** | **0.03** | **0.039** | 0.335 | **0.082** | **0.012** | **0.038** | 0.147 | 0.146 |
| Pigments | **0.092** | 0.769 | 0.234 | **0.036** | **0.05** | **0.077** | 0.316 | **0.066** | **0.041** | **0.052** | 0.358 | 0.324 |
| Polyphosphate degradation | **0.084** | 0.206 | **0.043** | **0.044** | **0.018** | **0.02** | 0.344 | **0.055** | **0.02** | **0.031** | **0.079** | **0.087** |
| Polyphosphate synthesis | **0.072** | 0.211 | **0.038** | **0.047** | **0.013** | **0.028** | 0.329 | **0.058** | **0.012** | **0.041** | **0.06** | **0.079** |
| S Reduction | **0.071** | 0.251 | **0.058** | **0.059** | **0.023** | **0.031** | 0.3 | **0.045** | **0.01** | **0.044** | 0.114 | 0.129 |
| Silicon | **0.038** | 0.335 | **0.095** | **0.055** | **0.016** | **0.028** | 0.511 | **0.059** | **0.058** | **0.038** | **0.048** | **0.031** |
| Sulfide Oxidation | **0.07** | 0.14 | **0.056** | **0.031** | **0.018** | **0.019** | 0.417 | **0.042** | **0.012** | **0.036** | **0.085** | 0.12 |
| Sulfur Oxidation | **0.063** | 0.288 | **0.045** | **0.023** | **0.007** | **0.009** | 0.338 | **0.025** | **0.013** | **0.025** | **0.075** | **0.079** |
| Tellurium | **0.083** | 0.336 | **0.073** | **0.043** | **0.018** | **0.02** | 0.431 | **0.058** | **0.018** | **0.051** | **0.09** | **0.084** |
| Aromatics | **0.081** | 0.155 | **0.047** | **0.035** | **0.019** | **0.012** | 0.344 | **0.052** | **0.004** | **0.024** | **0.073** | **0.096** |
| Chlorinated solvents | 0.144 | 0.98 | 0.693 | 0.163 | 0.212 | 0.412 | 0.296 | 0.21 | 0.204 | **0.094** | 0.762 | 0.672 |
| Polycyclic aromatics | 0.166 | 0.856 | 0.436 | 0.115 | 0.103 | 0.16 | 0.189 | 0.134 | **0.056** | **0.071** | 0.627 | 0.522 |
| Adenylylsulfate reductase | **0.047** | **0.087** | **0.02** | **0.026** | **0.011** | **0.012** | 0.352 | **0.029** | **0.008** | **0.021** | **0.046** | **0.065** |
| Virulence degradation | **0.068** | 0.287 | **0.083** | **0.048** | **0.027** | **0.046** | 0.279 | **0.077** | **0.015** | **0.037** | 0.151 | 0.132 |
| Halogenated compounds | 0.113 | 0.49 | 0.266 | **0.099** | **0.05** | 0.1 | 0.569 | 0.156 | **0.083** | **0.084** | 0.2 | 0.177 |
| Sulfite reduction | **0.075** | 0.214 | **0.066** | **0.038** | **0.018** | **0.017** | 0.436 | **0.042** | **0.01** | **0.034** | **0.078** | **0.074** |

*Significant differences (P<0.05) are indicated in bold.
